# Patients, Caregivers, and Healthcare Providers’ Experiences with COVID Care and Recovery across the Care Continuum: A Qualitative Study

**DOI:** 10.5334/ijic.6952

**Published:** 2023-06-16

**Authors:** Marina B. Wasilewski, Zara Szigeti, Christine L. Sheppard, Jacqueline Minezes, Sander L. Hitzig, Amanda L. Mayo, Lawrence R. Robinson, Maria Lung, Robert Simpson

**Affiliations:** 1 St. John’s Rehab Research Program, Sunnybrook Research Institute, Sunnybrook Health Sciences Centre, Department of Occupational Science and Occupational Therapy & Rehabilitation Sciences Institute, Temerty Faculty of Medicine, University of Toronto, CA; 2 St. John’s Rehab Research Program, Sunnybrook Research Institute, Sunnybrook Health Sciences Centre, CA; 3 Wellesley Institute, CA; 4 Musculoskeletal/STAR Rehab and Restorative Transitional Unit, St. John’s Rehab, Sunnybrook Health Sciences Centre, CA; 5 St. John’s Rehab Research Program, Sunnybrook Research Institute, Sunnybrook Health Sciences Centre, Department of Physical Medicine and Rehabilitation, Temerty Faculty of Medicine, University of Toronto, CA; 6 Department of Physical Medicine and Rehabilitation & Rehabilitation Sciences Institute, Temerty Faculty of Medicine, University of Toronto, CA

**Keywords:** COVID care quality, continuum of care, care transition, rehabilitation, qualitative

## Abstract

**Introduction:**

During the COVID-19 pandemic, discharge timelines were accelerated and patients were moved across the continuum of care, from acute to post-acute care, to relieve the strain in health system capacity. This study aimed to investigate the COVID-19 care pathway from the perspective of patients, caregivers, and healthcare providers to understand their experiences with care and recovery within and across care settings.

**Methods:**

A qualitative descriptive study. Patients and their families from an inpatient COVID-19 unit and healthcare providers from an acute or rehabilitation COVID-19 unit were interviewed.

**Results:**

A total of 27 participants were interviewed. Three major themes were identified: 1) The perceived quality and pace of COVID-19 care improved from acute care to inpatient rehabilitation; 2) Care transitions were especially distressing; and 3) Recovery from COVID-19 stagnated in the community.

**Conclusion:**

Inpatient rehabilitation was viewed as higher quality due to the slower paced care. Care transitions were distressing for stakeholders and enhanced integration between acute and rehabilitation care were suggested to improve patient handover. A lack of rehabilitation access led to recovery stagnating for patients discharged to the community. Telerehab may improve the transition to home and ensure access to adequate rehabilitation and support in the community.

## Background

The COVID-19 (COVID) pandemic declared on March 11th, 2020 has led to unprecedented mortality and morbidity. While a great deal of initial focus was on survival and acute care, clinical and research initiatives have now shifted to addressing the staggering disability faced by patients who survive a COVID infection. The majority of patients who are hospitalized and mechanically ventilated do not return to baseline at the time of discharge and require ongoing care to address a range of physical, cognitive, and psychosocial sequelae [1]. For many, this has entailed a complex recovery comprised of interactions with multiple care providers in various care settings before returning to the community [2,3]. Navigating the care continuum is challenging for patients and families in the best of times and the COVID pandemic has undoubtedly overwhelmed the normal process of patient care management and increased the difficulty of care transitions and patient follow-up [4]. Although care transitions can be optimized through the support of family caregivers, they are often poorly engaged in the process [5]. This gap in care was intensified by the COVID pandemic, where “no visitor” policies restricted the physical presence and involvement of family caregivers in care planning and coordination [6].

One of the hallmark pressures of the pandemic was the extraordinary surge in newly infected patients being hospitalized at one time, leading to strains in health system capacity across the continuum of care [7]. One way that this challenge was met in the acute care setting was by accelerating the discharge timeline to move patients who had recovered from the most acute phase of their illness to post-acute care (e.g., inpatient rehabilitation) in order to free up beds for subsequent surges [8,9]. Although this strategy facilitated the transfer of the “least sick” patients [8], it precipitated a number of fears and anxieties amongst patients, families, and healthcare providers (HCPs), and enhanced the risk of patient complications, readmission, and disconnected care [4]. It was also unclear during the early days of the pandemic if rehabilitation hospitals were prepared for incoming COVID patients, with many experiencing shortages in both personnel and resources [10,11]. Infection prevention and control measures further strained the accessibility of available therapies, services, and care providers. This was especially true in the community setting where outpatient and in-home rehabilitation services were suspended, resulting in many previously hospitalized COVID patients receiving little or no rehabilitation after an ICU stay [11].

The speed with which COVID patients transitioned across the care continuum coupled with limited family involvement and variable preparedness of healthcare settings, highlight the importance of taking a cross-continuum approach to investigations of COVID care. To date, no studies have explored the experiences of COVID patients who were hospitalized, received inpatient rehabilitation, and returned to the community. Further, studies have yet to integrate the perspectives of family caregivers and HCPs who were involved in this care process. For this reason, we investigated the COVID care pathway from the perspective of patients, caregivers, and HCPs (‘stakeholders’) with the goal of understanding their experiences with care and recovery within and across care settings.

## Methods

This paper draws on data from a study investigating the implementation and impact of COVID care within a hospital network comprised of an acute care and a nearby inpatient rehabilitation facility based in Toronto, Canada. We employed a qualitative descriptive approach, which entails a concise and descriptively rich analysis that remains true to participants’ own words [12,13]. Thus, it produces a data-near report that is representative of participants’ views, making it meaningful for key stakeholders and relevant for justifying actionable change [12,13].

### Participants and Recruitment

We recruited HCPs working in or supporting an acute or rehabilitation COVID unit by email using the hospitals’ COVID unit listservs. We recruited patients from an inpatient COVID rehabilitation zone between March-September 2020 and also obtained their caregiver’s information. Patients were referred by a member of their circle of care. Patients were eligible to participate if they were English speaking and cognitively able to provide consent. Caregivers were eligible if they were a friend or family member supporting a patient that met these criteria and were themselves English speaking and cognitively able to provide informed consent. We contacted 23 patients and caregivers, of which, n = 9 were not interested in participating and n = 4 could not be reached. Patient-to-provider ratios at the rehabilitation hospital vary according to the number of patients on a unit at a given time, with typical ratios being 1:4 for nursing and 1:7 for therapists. During the study period, the ratio of patients-to-providers in the COVID zone would have fluctuated but remained close to typical ratios.

### Data collection

This study was approved by the Research Ethics Board (REB) at Sunnybrook Health Sciences Centre. Informed consent was obtained prior to data collection. One trained qualitative researcher (SG) conducted all interviews via telephone or Zoom between August 2020 and February 2021 (See Table 1 for interview guide overview). Data were collected until saturation of ideas was reached. The interviewer and the research team were embedded within the inpatient rehabilitation hospital, and participants had no prior relationship with the interviewer and understood that the study goals were to explore stakeholder experiences with COVID care. Interviews ranged from 30 to 80 minutes, were audio-recorded, and transcribed verbatim. All identifying information was removed from the transcripts, which were uploaded to NVivo for organization and analysis. Sociodemographic information was collected from patients and family caregivers as well as clinical characteristics for patients. We also collected professional practice information from HCPs (e.g., profession, education, practice setting).

**Table 1:** Semi-Structured Interview Questions for Stakeholders.


STAKEHOLDER GROUP	INTERVIEW QUESTIONS

Patients	Could you tell me a little bit about your COVID-19 care journey? Could you tell me what happened when you arrived at [acute care site]? What was it like receiving care at [acute care site] for COVID-19? Overall, what went well during your stay at [acute care site] and what could have been improved? Can you tell me a little bit about your experience of preparing to leave [acute care site] to go rehab? How prepared did you feel for the transition to rehab? How did the transition go? What was it like receiving care at [rehab facility] for COVID-19? Overall, what went well during your stay at [rehab facility] and what could have been improved? How did you feel about getting ready to go home? How did the discharge to home go? After returning to home, did you feel like you had the necessary information and resources to support your continued recovery from COVID-19? Once you were home, what were your top concerns and needs?

Caregivers	Could you tell me a little bit about you and your loved one’s COVID-19 care journey? Could you tell me what happened when your loved one arrived at [acute care site]? What was it like to support your loved one while they were receiving care at [acute care site] for COVID-19? Overall, what went well during your loved one’s stay at [acute care site] and what could have been improved? Can you tell me a little bit about your experience as your loved one was preparing to leave [acute care site] to go rehab? How prepared did you feel for your loved one’s transition to rehab? How did the transition go? What was it like to support your loved one while they were receiving care at [rehab facility] for COVID-19? Overall, what went well during your loved one’s stay at [rehab facility] and what could have been improved? How did you feel about your loved one getting ready to go home? How did the discharge to home go? After your loved one returned to home, did you feel like you had the necessary information and resources to support their continued recovery from COVID-19? Once your loved one was home, what were your top concerns and needs?

HCPs	To start, could you tell me a little about your clinical role and work history? What were your thoughts and expectations when you first heard that a portion of [unit] was going to be converted into a COVID Zone? What were your top concerns, questions, and needs when you learned that you would be working in the COVID zone? How do feel your needs/questions/concerns were addressed by managers, administrators and other senior leaders? For managers/administrators/senior leaders: How did you address the needs/questions/concerns that staff had? What has the actual experience of working in the COVID Zone with COVID patients been like? What are your thoughts on the extent and quality of care that was delivered to COVID patients? Reflecting on your experience with working in the COVID Zone, what do you think the top successes were? Reflecting on your experience with working in the COVID Zone, what do you think the top areas for improvement were?


### Data analysis

We used an inductive thematic approach following the steps outlined by Braun and Clarke, whereby data were deconstructed into isolated fragments followed by reconstruction into overarching themes that describe the higher-level messaging in the data [14]. Two independent researchers (ZS and SG) completed the coding process and three additional researchers (CS, RS, MBW) participated in the thematic analysis.

### Rigor

Analytic rigor was enhanced by triangulating between multiple individuals throughout analysis, having regular team meetings, and exercising reflexivity (discussing and journaling the study team’s own biases and experiences that may influence data interpretation). We also adhered to the COREQ reporting guidelines [15].

## Results

In total, we interviewed 27 participants for this study, which included 10 patients, 5 caregivers and 12 HCPs (See Table 2 for patient and caregiver characteristics). Of the 12 HCPs, n = 3 worked in the acute care setting while the remaining n = 9 worked in the inpatient rehabilitation setting. HCPs were occupational therapists (n = 3), patient care managers (n = 2), registered nurses (n = 2), medical department heads (n = 2), collaborative practice leaders (n = 2), and a pharmacist (n = 1). All HCPs (n = 12) reported a graduate level education.

**Table 2:** Patient and Caregiver Sociodemographic Characteristics (n = 15).


CHARACTERISTIC (MEAN, SD)	PATIENT (n = 10)	CAREGIVER (n = 5)

Age in Years	62.8 (17.9)	60.2 (4.3)

Length of Stay in Rehab in Days	12.4 (1.8)	n/a

**CHARACTERISTIC (n, %)**	**PATIENT (n = 10)**	**CAREGIVER (n = 5)**

Positive COVID Status Upon Rehabilitation Admission	4 (40)	n/a

Gender		

Male	2 (20)	2 (40)

Female	7 (70)	3 (60)

Did not disclose	1 (10)	–

Birth Country		

Canada	3 (30)	3 (60)

China	2 (20)	–

Guyana	1 (10)	–

Nigeria	1 (10)	1 (20)

Philippines	2 (20)	1 (20)

USA	1 (10)	–

Ethnicity		

Black	1 (10)	1 (20)

Chinese	2 (20)	–

Filipino	2 (20)	1 (20)

Indian	1 (10)	–

South Asian	1 (10)	–

White	3 (30)	3 (60)

Marital Status		

Married	2 (20)	5 (100)

Widowed	4 (40)	–

Single	2 (20)	–

Common law	1 (10)	–

Did not disclose	1 (10)	–

Education		

Some high school	3 (30)	–

Completed college	3 (30)	2 (40)

Completed university	3 (30)	3 (60)

Graduate program	1 (10)	–

Yearly Income (CAD)		

10,000–19,999	2 (20)	–

20,000–29,999	2 (20)	–

30,000–39,999	2 (20)	1 (20)

40,000–49,999	–	1 (20)

50,000–59,999	–	–

60,000–69,999	1 (10)	2 (40)

Did not disclose	3 (30)	1 (20)


Across patient participants, recollection of their early acute care experiences was limited due to the severity of their illnesses. Many patients explained that they were ventilated and/or in a coma and stayed in the intensive care unit (ICU) for some period. As one participant described: “*I was in intensive care for three days…I cannot remember one, one thing. I can’t remember anybody coming near me. I can’t remember a doctor. I can’t remember a nurse. That whole three days is three days out of my life that I cannot remember*.” (PT01). According to patients, they spent anywhere between one to eleven weeks in the acute care setting before being transferred to the inpatient rehabilitation hospital. The health challenges they encountered varied but included severe debility and deconditioning, walking and speech difficulties, breathing problems, as well as pneumonia. Additional patient demographic and rehabilitation information can be found in Table 2.

Most explained.

### Theme 1: The perceived quality and pace of COVID care improved from acute care to inpatient rehabilitation

Participants explained that throughout the pandemic there were specific circumstances and policies that impacted the ability to provide care and address patient needs within a healthcare institution. Firstly, the novelty of the COVID virus generated widespread fear and anxiety about *“the unknown”* amongst patients, HCPs, and caregivers alike (HCP04, PT06, PT11, CG04). For everyone, but especially patients, this heightened the need for informational and emotional support. Patients and caregivers explained that they would typically turn to their healthcare team for information regarding their condition; however, the novelty of COVID meant that even HCPs felt they lacked the usual level of insight and knowledge regarding patient condition, intervention, and outcomes. Consequently, HCPs described feeling *“not so confident to answer all their [patients’] questions”* (HCP01).

COVID also created an environment where resources were depleted, rendering the entire healthcare system—especially acute care settings—scarcely able to accommodate the high and constant influx of COVID patients. HCPs described that public health guidelines mandated they operate on a *“get in and get out type of attitude”* (HCP12) to minimize their risk of exposure, so they did not “*really want to spend any extra time in [patients’ rooms] than I had to”* (HCP10). Acute care was thus viewed as a particularly challenging environment where there was minimal time to *“do anything but run in and out of the room”* (HCP11). The rushed nature of care and constrained ability of HCPs to provide high-levels of support and information was felt amongst patient participants who described the acute care setting as *“unsettling”* (PT06), *“difficult”* (PT14), and a place where they were constantly left *“wondering why”* (PT06).

In inpatient rehabilitation, HCPs still felt pressure to minimize their exposure to COVID patients, which sometimes led to patients not being provided with therapy entirely because *“some people didn’t feel like the exposure of other people is worth it. Like really, do patients need to be seen for a ten minute treatment?”* (HCP01). Due to physical distancing requirements, the provision of comprehensive rehabilitation beyond the confines of patients’ rooms was also difficult. Therapy predominantly involved *“keeping [patients] in [the COVID area] of the unit. They couldn’t go to the gym, they couldn’t be walking all the halls for exercise, and they couldn’t be doing all these other things that they would normally do”* (HCP04). The detriments of these constraints was recognized by HCPs who acknowledged that *“therapy in the room is not as good as therapy in the gym, but we didn’t really have a choice”* (HCP01).

Despite the constraints and challenges expressed by rehabilitation HCPs, patients and caregivers overwhelmingly viewed the inpatient rehabilitation environment as more responsive to psychosocial needs and one where patients were *“treated like a person”* (CG07) and HCPs *“took the time”* (PT06) to reassure and address patients’ concerns. In fact, some patients described HCPs as “*so kind and nice in rehab, they were like family”* (PT01). This was in stark contrast to patients’ perception of acute care, where they felt HCPs distanced themselves from them to avoid contracting COVID. A key difference between care settings was that rehabilitation HCPs expressed and enacted fears and concerns around infectivity less explicitly, leading patients to feel more *“at home”* (PT05) and *“warm”* (CG07) in the rehabilitation setting and more *“comfortable with the nurses”* (CG04). HCPs themselves also described the rehabilitation environment as one with *“less patients and so we had more time to spend with them”* (HCP10) and thus were able to do *“the most healthy nursing I’ve done in years” (HCP08)*.

### Theme 2: Care transitions were especially distressing

Acute care HCPs felt forced to ‘triage’ patients by quickly moving them across the care continuum to *“get [them] home as quickly as possible to make room for the expected surge of COVID patients”* (HCP04). HCPs recognized that this problematically involved *“taking patients who maybe weren’t totally ready for rehab from [HOSPITAL] to make room for new patients coming in”* (HCP04). Despite the fact that some HCPs *“got some assistance”* (HCP06) from redeployed clinicians to better-support patients during transitions, patients and caregivers still felt *“like I was just rushed out* […] *I just felt I wasn’t ready to leave there”* (PT06). One patient further explained that it would have been helpful to have a rehabilitation HCP “*come [to acute care] and say ‘I’d like to introduce myself, I’m from [REHAB]’, and I could ask my questions and get some answers, I would have felt completely different” (PT06)*.

The sentiments of feeling unready and hurried from acute care to rehabilitation were echoed as patients prepared to be discharged from inpatient rehabilitation to home. Once patients started displaying functional gains, they were told *“I’m doing well, so then [DOCTOR] said we’re going to discharge you tomorrow. I was thinking I’d be there longer”* (PT14). Caregivers shared the sentiments of wishing their loved ones had more inpatient rehabilitation and highlighted how *“more physio would’ve been great”* (GC04) and that *“whatever she got there, it just wasn’t enough”* (CG03). Patients described that they felt unready to go home for a number of reasons including worrying “*when I go home, who’s going to cook for me, bathe me, stuff like that?*” (PT18) and also feeling *“not quite steady enough to go home”* (PT14). Once home, patients continued to feel unprepared as they still experienced limitations, such as a *“lack of confidence in my walking at home”* (PT15). Caregivers also highlighted their worries about their loved one transitioning home during a period where COVID conditions limited home care, voicing concerns about *“what type of care she’d get at home”* (GC03). While HCPs agreed that patients and caregivers were distressed about the transition to home, they attributed a portion of those sentiments to a fear of *“infecting other members of the family when they’re home. [Patients and caregivers] asked…do they need to isolate? Or what sorts of things do they need to protect themselves?”* (HCP06). Despite the challenging environments and difficulties associated with transitions, patients still described a strong feeling of *“excitement to go home”* (PT05, PT11) and eagerness to *“be with my family”* (PT05). Some patients went as far as to decline additional therapy in order to speed up the transition to home as they were longing to be reunited with their families.

### Theme 3: Recovery from COVID stagnated in the community

Despite the excitement to return home and a strong urge to *“get back to normal life”* (CG07, GC10), patients and caregivers found that once they were discharged, there was nowhere for them to go to continue receiving care that would enable them to progress in their recovery journey. Without adequate community-based support, patients’ recovery was described as being *“limited in a way”* (CG04). This translated into feelings of stagnant recovery at home and in the community.

Participants explained that COVID infectivity and related public health guidelines (i.e., stay at home orders and physical distancing) led to the discontinuation of outpatient and community-based supports that are typically provided to enable ongoing recovery at home following injury or illness. HCPs shed light on the lack of community based supports for patients, including how *“a lot of the homecare services were declining patient service at this time. If you had a diagnosis of COVID positive, we weren’t able to send these people home because they wouldn’t get their homecare services” (HCP05)*. This meant that COVID patients struggled to find physiotherapists, occupational therapists, and personal support workers to *“come into the home and help me, or bathe me, or even shopping, things like that”* (PT18). Even when patients were no longer believed to be infectious, there were still tremendous difficulties with accessing home-based care because *“[community workers] didn’t want to get COVID, plain and simple […] [community service] called us back saying they didn’t want to go into people’s homes, regardless of illness status”* (HCP10). Not only were patients struggling to access home-based care that would help them accomplish their activities of daily living, but there was *“a complete loss of out-patient services”* (HCP12), meaning that patients were unable to go to healthcare organizations to receive the services they knew would enable their continued physical recovery.

Without home and community-based supports, there was an additional element of stress and worry because *“patients and caregivers feared that we would send them home with no help”* (HCP05). In order to accommodate for COVID circumstances and limited home-based supports, rehabilitation HCPs adapted discharge planning to include *“workout sheets that I was told to do at home from my [in patient] physiotherapist”* (PT05), and *“phone numbers of physios that I called”* (PT17). Patients quickly realized, however, that these resources were not sufficient to facilitate ongoing recovery as patients were rarely able to get into contact with community-based healthcare services. If patients did reach these organizations, the wait to receive the services was extremely long, with some individuals being *“placed on a months-long wait list”* (PT18). Conversely, when services could be obtained, participants felt that they were offered far too infrequently, with some patients highlighting how *“coming once a week [to do my sponge bath] isn’t enough”* (PT14). Given the delays and decreased frequency of at-home services, many patient participants took elements of their recovery into their own hands, explaining that they *“couldn’t wait more than the three weeks, and at that point it was too late […] so I just did [rehab exercises] myself”* (PT15), which sometimes entailed Internet searches for advice about ‘deconditioning’ and watching online videos of exercises.

## Discussion

Our study explored the experiences of patients, family caregivers, and HCPs with COVID care and recovery across the continuum of care. The crisis conditions evoked by the COVID pandemic challenged various aspects of patients’ recovery across the continuum of care. Circumstances such as the fear surrounding COVID infectivity, HCPs receiving directives to minimize contact and exposure risk, restricted visitation policies, and a general paucity of information surrounding COVID all created heightened patient needs for information, transparency, communication, and support. However, these same circumstances that precipitated greater patient need also rendered the healthcare system pragmatically and logistically unable to adequately meet those needs. Our findings highlight that the COVID pandemic exacerbated existing challenges within the healthcare system, which complicated recovery across the care continuum. Three themes were identified and elucidated that: 1) the inpatient rehabilitation setting, compared to acute care, was slower-paced and led to care that was perceived as higher quality by patients; 2) care transitions were stressful, with patients and caregivers feeling unprepared to transition from acute care to inpatient rehabilitation and then to home; and 3) depleted resources and COVID restrictions made community-based recovery difficult.

### The importance of the inpatient rehabilitation setting in the COVID care pathway

Our study demonstrates the important role that inpatient rehabilitation plays in the continuum of COVID care. From the perspective of enhancing patient flow throughout the continuum of care, inpatient rehabilitation served as a ‘pressure release valve’ for the acute setting by creating additional care opportunities for COVID patients who were no longer in the most acute stages of illness but could not yet return home unsupported. Our findings suggest that while patients felt ‘rushed’ out of acute care, they viewed inpatient rehabilitation as far less ‘fast-paced’, which translated into perceptions of care being higher quality. Notably, this was in spite of the fact that HCPs themselves felt that the extent and quality of rehabilitation care provided was not as good as it may typically be outside of pandemic conditions. By capturing experiences from multiple stakeholders’ perspectives, we were able to identify and reflect on this important dichotomy in views. It is possible that while HCPs measure the success and impact of their care through the content and quality of therapies provided, patients themselves place greater importance on the relational aspects of that care. It is known that the relationship between rehabilitation HCPs and patients is a key enabler of both positive experiences and improved patient outcomes, where positive relationships are supported by caring, empathic, and respectful behaviors from HCPs [16]. These were the types of behaviors patients in our study witnessed from their HCPs and left the most prominent and positive impact on patient and caregiver perceptions of care, lending support to research that has demonstrated HCPs’ skills and attitudes to be highly linked with patient satisfaction [17]. Our study underscores that especially during a crisis such as the COVID pandemic, ensuring high quality interactions between patients and HCPs is imperative to enhancing the patient experience and promoting patient recovery—both within the inpatient rehabilitation setting and across the continuum of care. As has been suggested by other studies [16] and echoed by our findings, important aspects of relational care include caring and empathic behaviors from HCPs and a willingness on the part of HCPs to engage with patients with interest, to understand their needs, and address their concerns.

### Optimizing transitions across the COVID care continuum

Most studies investigating the ways that care transitions were impacted by the COVID pandemic have centered on non-COVID populations (e.g. mental health, stroke, dementia) [18,19,20]. Studies that have focused on the COVID care continuum have disproportionately considered decision-making pathways, screening, and care processes [21,22]. In turn, this body of evidence has yet to consider care transitions from the perspective of COVID patients, family caregivers, and HCPs. Presently, the overarching trend in healthcare is to reduce costs by reducing LOS for patients [23,24]. Our data suggests that participants felt that LOS was shorter than desired during acute COVD care and perceived this to be due to the need to free beds for additional patients. However, this shortened LOS was not adequately coupled with enhanced patient preparation for earlier discharge. As reported in other studies, patients and caregivers in the current study felt unprepared to transition from acute care to inpatient rehabilitation and lacked the understanding and education that might ease this transition [23].

The COVID pandemic generated an unprecedented demand for high quality transitional care while simultaneously precipitating conditions that undermined the core components of transitional care: (1) engaging patients and family caregivers; (2) educating patients and their caregivers; (3) managing complex health and social needs; (4) promoting well-being; and (5) assuring continuity and accountability for care [25]. Our findings point to several ways that these components can be enhanced in order to optimize COVID care transitions:

1. Enhance integration between acute and rehabilitation care settings: Many patients in our study felt unprepared to transition between environments and much of this was due to a lack of information about what the next steps of recovery entail. Other research has noted that this is a common issue beyond COVID, with patients not being educated about what to expect from the inpatient rehabilitation environment [26,27]. As one patient in our study suggested, it may be beneficial to have a representative from the inpatient rehabilitation hospital meet the patient and family prior to transfer. This has the potential to enhance continuity of both information and relationships and advance an integrated care approach that promotes the inclusion of rehabilitation professionals early on in the continuum of care and recovery. Previous research has suggested that early physical medicine and rehabilitation consultations can decrease LOS in acute care, improve patients’ post-discharge function, and help guide patients to the most appropriate care for their needs [23,28]. Given the rapid and widespread use of telehealth at the outset of the pandemic, this modality may represent an opportune avenue to initiate early linkages with rehabilitation HCPs to better prepare patients and families for the next step in the recovery journey.
2. Address multi-faceted health and social care needs: Our findings demonstrated that the COVID pandemic exacerbated common challenges with transitioning home after inpatient rehabilitation. Many of the challenges that emerged in participant narratives highlighted the importance of not only addressing patients’ functional recovery and physical safety, but also considering the complexity of patient and families’ health and social needs. One aspect unique to COVID care was the challenge faced by patients and caregivers to ensure infection prevention and safety upon return to home. This was recognized by HCPs in our study to be an added source of stress for patients and families at the time of discharge and emphasizes the importance of addressing both the physical and psychological aspects of patient and caregiver safety [24]. Research with other rehabilitation populations has noted that traditional discharge planning over-emphasizes functional status with little attention paid to the psychosocial aspects of recovery, which is detrimental to patient care since discharge readiness’s hinges not only on physical ability but psychological ability as well as availability of family and community support [24]. In turn, clinical pathways for COVID patients would benefit from added planning and considerations for ‘psychological safety’ in order to enhance discharge readiness—especially back to the community.

### Recovery beyond the walls of the hospital

As others have pointed out, while discharge to home is often an optimal outcome after hospitalization, home health care was not able to provide the necessary support for those recovering from COVID-19 [11]. Coupled with widespread closures of outpatient rehabilitation services, patients were not able to access rehabilitation services outside of the inpatient setting [11]. This was certainly the experience of patients in the current study where the prevailing sentiment amongst participants was that this lack of access hindered ongoing recovery once patients returned to home. This appears to confirm existing concerns that the closure of rehabilitation clinics and lack of home care support may lead patients to recover more slowly [7] and thus render this a population with unique post-discharge needs [1]. One avenue for augmenting rehabilitation support and recovery for patients outside of the walls of the hospital is through telerehabilitation (telerehab). Despite the widespread use of telerehab in the wake of the COVID pandemic [29,30,31], its application for COVID patients themselves has been comparatively limited. One of the few studies that has explored telerehab for COVID patients focused on the inpatient rehabilitation setting [32], further highlighting that telerehab’s potential for supporting community-based recovery has yet to be realized. Although telerehab is an ideal way to provide post-discharge assessments of patient needs and to deliver key interventions [1,33], none of the participants in our study made mention of any virtual care options post-discharge, underscoring a significant gap in care and area for future research.

### Strengths and limitations

A notable strength of this study is the exploration of patients, caregivers, and HCPs’ lived experiences across the continuum of care. We were successful in achieving robust sample sizes for patient and HCP stakeholder groups; however, family caregivers could have been better-represented in the sample. We achieved diversity in patient ethnicity and HCPs’ clinical roles. However, we were not able to include HCPs from the community setting and all participants in our study were English-speaking, thus, our findings are limited in their transferability to community care practitioners and linguistically diverse individuals. Our study is reflective of one hospital network and clinical pathway (i.e. acute care to inpatient rehabilitation to home). Thus, findings may not be transferable to other pathways that patients and families may take for COVID care. While we were able to access information about patients’ rehabilitation care through a rehabilitation database, we did not have access to patients’ acute care charts and thus could not report on acute care LOS, patients’ acute health status and acute care interventions received. In turn, our study is limited in contextualizing participant experiences and patient-provider interactions based on acute care circumstances. Finally, our study represents a ‘snapshot’ of a specific period during the pandemic, and thus the perspectives of stakeholders may change or evolve as policies and procedures are modified based on emerging knowledge of COVID-19 and as the pandemic evolves with new variants, treatments, and preventive measures (e.g. vaccinations).

## Conclusion

The COVID pandemic exacerbated existing challenges within the healthcare system, thereby complicating recovery across the care continuum. Inpatient rehabilitation was viewed as higher quality due to the slower pace and more relational care received compared to the acute setting. Care transitions across the continuum were distressing for patients and caregivers. Upon return to the community, a lack of rehabilitation access led to recovery stagnating. Our findings highlight that inpatient rehabilitation plays an important role in the COVID care continuum by providing patients with a period of slower paced care to support psychosocial recovery. Greater integration of acute and inpatient rehabilitation settings can ameliorate care transitions, while telerehab has the potential to improve the transition to home and ensure that patients and caregivers have access to adequate rehabilitation and support in the community.
